# Effect of Substrates' Compliance on the Jumping Mechanism of *Locusta migratoria*

**DOI:** 10.3389/fbioe.2020.00661

**Published:** 2020-07-06

**Authors:** Xiaojuan Mo, Donato Romano, Marco Miraglia, Wenjie Ge, Cesare Stefanini

**Affiliations:** ^1^School of Mechanical Engineering, Northwestern Polytechnical University, Xi'an, China; ^2^Sant'Anna School of Advanced Studies, The BioRobotics Institute, Pisa, Italy; ^3^Department of Excellence in Robotics & A.I., Sant'Anna School of Advanced Studies, Pisa, Italy; ^4^Healthcare Engineering Innovation Center (HEIC), Khalifa University, Abu Dhabi, United Arab Emirates

**Keywords:** locust, *Locusta migratoria*, substrate, compliance, jumping mechanism, jumping performance

## Abstract

Locusts generally live and move in complex environments including different kind of substrates, ranging from compliant leaves to stiff branches. Since the contact force generates deformation of the substrate, a certain amount of energy is dissipated each time when locust jumps from a compliant substrate. In published researches, it is proven that only tree frogs are capable of recovering part of the energy that had been accumulated in the substrate as deformation energy in the initial pushing phase, just before leaving the ground. The jumping performances of adult *Locusta migratoria* on substrates of three different compliances demonstrate that locusts are able to adapt their jumping mode to the mechanical characteristics of the substrate. Recorded high speed videos illustrate the existence of deformed substrate's recoil before the end of the takeoff phase when locusts jump from compliant substrates, which indicates their ability of recovering part of energy from the substrate deformation. This adaptability is supposed to be related to the catapult mechanism adopted in locusts' jump thanks to their long hind legs and sticky tarsus. These findings improve the understanding of the jumping mechanism of locusts, as well as can be used to develop artifact outperforming current jumping robots in unstructured scenarios.

## Introduction

Many animals can move on several kinds of substrates. A substrate can include biotic or abiotic materials. The physical interaction between an animal and a particular substrate can be considered as a complex adhesion and contact problem in which two bodies are involved. Both have various geometrical, mechanical and chemical properties (Gorb and Gorb, 2009). The roughness, compliance (or inverse, stiffness), Young modulus, humidity and even viscosity of a substrate could be key parameters that impact the contact and the strategies an animal adopts to move on that particular substrate. These strategies have also inspired roboticists to enhance the locomotion ability and environmental adaptability of their robots (Sitti and Fearing, 2003; Menon et al., 2004; Unver et al., 2006; Sintov et al., 2011; Lee, 2018).

The impact of substrates' roughness on animals' locomotion abilities and the grasping mechanisms of different animals have been thoroughly studied. The grasping mechanisms of animals can be divided into: (*i*) dry adhesion, used by geckos (Autumn, 2007; Zhou et al., 2013; Cutkosky, 2015); (*ii*) wet adhesion, used by frogs (Persson, 2007); (*iii*) glues, used by mussels (Lee et al., 2006), starfishes (Hennebert et al., 2014) and sea cucumbers (Flammang et al., 2002); (*iv*) suction, used by octopus (Tramacere et al., 2014); (*v*) interlocking, used by leopards, squirrels (Cartmill, 1974), insects (Pattrick et al., 2018) and birds (Gorb, 2008). Many of these grasping strategies have been widely adopted in robots' design (Li et al., 2016). Dry adhesion with one billion spatulas on geckoes' toes (Autumn, 2007) exploits van der Waals interaction forces and inspired climbing robots on smooth substrates (Sitti and Fearing, 2003; Menon et al., 2004; Unver et al., 2006), while compliant feet and rigid claws gave inspiration to the design of climbing robots on rough substrates (Sintov et al., 2011; Xu et al., 2016; Ji et al., 2018; Lee, 2018).

Locusts can adapt to substrates of various roughness, thanks to a combined grasping mechanism consisting of rigid claws that generate mechanical interlocking on rough substrates, and adhesive pads for vacuum adhesion on smooth substrates (Goodwyn et al., 2006; Wang et al., 2009, 2015; Mo et al., 2019). This particular characteristic can be considered as a sort of morphological intelligence, which makes locusts capable of dealing with a wide variety of substrates, even much different from each other, avoiding slipping phenomena in both jumping and landing phase (Woodward and Sitti, 2018). Most animals, instead, have developed specific adaptation strategies toward a particular type of substrate. Leafhoppers can jump successfully from smooth surfaces by increasing the contact area between pads and substrate (Clemente et al., 2017), while froghoppers can jump from smooth plant surfaces by piercing them with sharp spines (Goetzke et al., 2019). Based on these grasping strategies adopted by animals, researchers added spines (Lee and Fearing, 2015) and adhesive pads to the feet of running (Lee and Fearing, 2015) and jumping robots (Lee et al., 2016, 2018) in order to increase performances on different substrates.

Even if substrates with similar roughness values are considered, many other different aspects exist. Among them, the substrates' compliance level is the main one, directly affecting the contact dynamics (Cannell and Morgan, 1987). Nowadays, the effect of substrates' compliance on animals' locomotion have been object of a few studies (Demes et al., 1995; Thorpe et al., 2007; Gilman et al., 2012; Ribak et al., 2012; Gilman and Irschick, 2013; Astley et al., 2015; Knight, 2015), although it has relevant importance in natural environments. Therefore, the question is still open: how can animals move on substrates of different compliance? How are the locomotion performances influenced by the substrates' compliance?

The effects of natural substrates on the jumping height of click-beetles have been tested and, concerning the particular effect of substrates' compliance, also experimental tests on artificial substrates have been performed (Ribak et al., 2012). Coincidence between theoretical results coming from a mathematical model and experimental results has been found, both demonstrating that click-beetles do not have the ability to adjust their jumping dynamics according to the substrate's compliance level; they simply use maximum power to jump from any kind of substrate, ignoring the energy lost in the deformation of the substrate. Click-beetles even abolish the practice of jump when they are on very soft surfaces, such as cotton wool (Evans, 1972, 1973). Click-beetles also use jumping to straighten up if landed on their back and to control the body asset during the takeoff phase thanks to that small torque exerted on the ground in the contact point with feet (Ribak and Weihs, 2011). The ability of the beetle to complete the minimal rotation, necessary for righting itself, is limited by the energy attenuation observed when jumping from leaves (Ribak et al., 2012). Similar situations happen in doves *Geopelia cuneate*: their takeoff velocity is negatively impacted by higher levels of substrates' compliance, while their landing velocity is less impacted and landing stability problems are properly managed by wings and tail. In addition, free-living doves avoid the negative impacts of compliance by selecting stiffer perches (Crandell et al., 2018).

For lizards, even if they are normally found on compliant perches, during basking, foraging or other kinds of activities, the effect of substrates' compliance is dramatic and they avoid to jump from highly compliant perches (Gilman and Irschick, 2013). Experimental tests showed that lizards leave compliant perches before recoil of the perches occurs, and higher levels of perch compliance correspond to lower jumping distances and takeoff speeds, likely because of the kinetic energy lost in the flexion of the perch (Gilman et al., 2012). This effect is strongly connected to the mass of lizards: in bigger lizards (mass > 3 g), the influence is much more significant both on velocity and distance. Perch compliance also causes physical instability during the jump, particularly in small lizards since their tail remains in contact with the perch during the jump (Gilman and Irschick, 2013). Grabar et al. found that the jumping kinematics and performance of two tested species of gecko, the *Correlophus ciliatus* and *Rhacodactylus auriculatus*, are scarcely impacted by the substrate geometry (Grabar et al., 2016), while mass has a positive effect on jumping distance and takeoff velocity in *C.ciliatus*. It is demonstrated that lizards own the ability to jump in order to overcome the environmental challenges and obstacles.

In Cuban tree frogs (*Osteopilus septentrionalis*), the situation is different (Astley et al., 2015; Knight, 2015). Their jumping performances are nearly not affected by perches' compliance variability; just the takeoff velocity is a little bit penalized on compliant surfaces. In fact, during the recoil phase, these frogs are able to regain part energy lost because of the perch compliance. This is obtained thanks to long legs and sticky toes, that enable these frogs to keep longer contact time with perches (Astley et al., 2015).

It is well-known that compliance of perches is exploited by orangutans to reduce the energy costs when they pass from a perch to another (Thorpe et al., 2007). Energy consumption in tree swaying is found to be less than half of jumping, and an order of magnitude lower than in tree descending activity, walking or climbing the tree-trunk (Thorpe et al., 2007). Previously, primates were considered to increase energy costs when crossing gaps between compliant perches (Alexander, 1991; Demes et al., 1995). Although wild leaping primates are hypothesized to share similar mechanical mechanisms, gibbons use different leaping strategies: slower orthograde leaps on soft substrates and more rapid pronograde leaps on stiffer substrates in order to minimize perch deflection (Channon et al., 2011). Although both leaping strategies did not show energy recovery, gibbons are able to adjust their leap biomechanics in order to counterbalance the negative effect of the substrates' compliance (Channon et al., 2011). The locomotion performances of human beings on substrates of different compliance are thoroughly studied (Zamparo et al., 1992; Kerdok, 1999; Kerdok et al., 2002; Moritz and Farley, 2003, 2005; Coward and Halsey, 2014). Humans can adjust the position of the gravity center by varying legs' configuration to compensate for moderate changes in surface stiffness, during both hopping in place and running. This requires increased muscle activation, around 50%, on the softest surfaces with respect to the stiffest ones (Moritz and Farley, 2005).

As concerns locusts, no significant differences in jumping performances have been highlighted by considering substrates of different roughness (Mo et al., 2019; Wan et al., 2020). Locusts' habitat includes plants stem, leaves, dry branches and structured pavements. Among these kinds of substrate, the characteristics of roughness and compliance are much heterogeneous. It is very appealing to study the effect of substrates' compliance on locusts' jump performances and their evolved strategies. Based on authors' limited knowledge, there is no similar research on locusts and even few researches on insects in general (Evans, 1972, 1973; Thorpe et al., 2007; Ribak et al., 2012).

In this paper, performances and peculiar characteristics of *Locusta migratoria* jumping are tested and assessed on three kinds of substrates, mainly differing for the level of compliance. A dedicated test bench was fabricated, in order to support the three kinds of substrate. The experimental procedures and guidelines are exhaustively described. Firstly, results are analyzed by means of a statistical generalized linear model, then they are illustrated with the support of histograms and finally they are deeply discussed highlighting the specific jumping strategies of *L.migratoria*. In Appendix, a mathematical model of the substrates dynamics is reported as starting point for future works in which experimental results may be supported and integrated with theoretical ones.

## Materials and Methods

### Animal Rearing

*L. migratoria* adults were reared at the BioRobotics Institute, Scuola Superiore Sant'Anna (Pisa, Italy). Experiments were conducted under laboratory conditions in March 2019. All tested locusts were reared in different cylindrical transparent plastic boxes (50 cm diameter and 70 cm length) with a 16:8 (L:D) h photoperiod at 25 ± 1°C, 40 ± 5 % RH. Temperature and RH conditions were kept constant during experiments. A total of 226 adult locusts were tested, recording their jumps with a high-speed camera. During the whole experimental period, the health of each locust was constantly checked, and locusts were fed with wheat, fresh vegetables and water. The experiments were carried out using healthy locusts with no injuries (e.g., no damaged legs, wings or antennae). All the locusts were weighed with a 0.01 g precision balance. The dimensions of the main physical features (i.e., body, femur, tibiae and tarsus length) were measured by means of 0.01 mm precision caliper.

### Experimental Procedure and General Observations

Three kinds of substrates with different compliance were used to test the effect of substrates' compliance on locusts jumping performances. Silicone membranes were used to test the jumping performances of locusts on compliant substrates. One layer of silicone membrane with 0.2 mm thickness forms the most compliant substrate, and three layers of silicone membranes with thickness of 0.6 mm form another less compliant substrate. From now on ward, they will be referred to as “most compliant substrate” and “compliant substrate.” The rigid substrate, instead, consists of a 3 mm thick aluminum plate. These three experimental substrates were selected in order to simulate locusts' jump from three types of natural substrates which locusts often have to deal with: aluminum plate corresponds to stiff branches, three layers of silicone simulate the behavior of stems and one layer of silicone simulates compliant leaves. The authors of the present work could a priori imagine different scenarios regarding locusts' jumping performances, depending on the level of adaptation of locusts to substrates of different compliance: *i*) in case of total adaptability, nearly no differences would have been detected in jumping performances from one substrate to another; *ii*) in case of poor adaptability, a significantly decreasing trend in performances would have been detected passing from the rigid substrate to the compliant one and then from the compliant to the most compliant; *iii*) in case of intermediate adaptability, performances would have been likely decreased a little from the rigid substrate to the compliant one, while there would have been much consistent deterioration of performances by passing from the compliant substrate to the most compliant.

**Figure 1:**
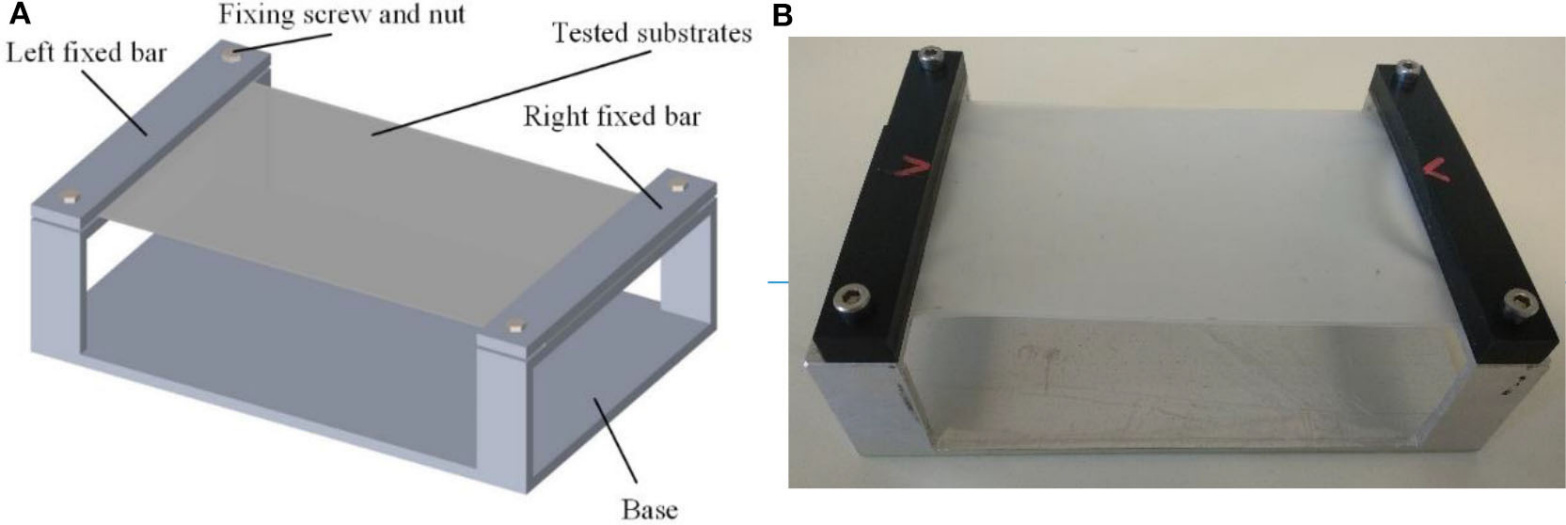
Test bench illustration. **(A)** Test bench 3D model **(B)** Fabricated tested bench.

In order to support the substrates, a dedicated test bench was fabricated as much big as possible compatibly with the high-speed camera field of view. The aim was to obtain the maximum possible flexibility in the center of the substrate (in particular for compliant and most compliant substrates), limiting at same time the influence of the external constraints. The in-plane dimensions of substrates are 120 × 80 mm. As shown in Figure 1, the silicone membrane substrates were fixed on an aluminum support by means of two fixing bars, hold in place by two couples of screws. The aluminum plate consists of the rigid substrate, once the test bench is flipped.

The test bench was placed inside a 30 × 50 × 40 cm foam box, whose front side was made of transparent acrylic screen and the back was lined with filter paper (Whatman no. 1). Locusts were stimulated using a transparent soft plastic bar, to elicit the maximum “escape jump.”

Roughness of aluminum and silicone were measured by means of a roughness meter (ZEISS/TSK, Zeiss SURFCOM 130A). Data are reported in Table 1 together with the mechanical properties. Based on previously results, published by the authors of this work (Mo et al., 2019), there is no significant difference on locusts' jumping performances if different values of substrate's roughness are considered. This is due to the combined action of rigid claws and adhesive pads, that ensures stable contact between tarsus and ground. Therefore, the roughness difference between aluminum (*Ra* = 0.113±0.004 μ*m*) and silicone (*Ra* = 0.525±0.027 μ*m*) was not taken into account in the conducted experiments and results analysis.

**Table 1:** Roughness values and mechanical properties of the two types of substrates used in the experiments.

	**Tested length (mm)**	**Ra (um)**	**Rz (um)**	**E (MPa)**	**ν (pure number)**
Membrane	3.000	0.525 ± 0.027	4.076 ± 0.319	~5	~0.5
Aluminum	3.000	0.113 ± 0.004	1.345 ± 0.211	7.2 × 10^4^	0.334

*Ra, roughness value; Rz, average of the ten highest peaks and the ten deepest valleys, E, young's modulus, ν, poisson's ratio*.

Each locust executed 5 jumps on each kind of substrate and was recorded each time. The jumps were interspersed by 10 min to allow the locust to recover totally between two consecutive jumps. Nominally, jumps are perpendicular to the axis of the camera. Jumps deviating more than 15° with respect to the plane perpendicular to the axis of the camera lens were let out in order to limit the difference between the actual and perceived takeoff angle (Baker and Cooter, 1979). A HotShot 512 SC high speed video camera (NAC Image Technology, Simi Valley, CA, USA), with maximum frame rate up to 200, 000 fps, was used to record the takeoff phase videos at a rate of 1000 fps (Romano et al., 2018, 2020; Mo et al., 2020). Sequential images with a resolution of 512 × 512 pixels were stored in the camera internal memory, and then downloaded for data analysis. The area where tested locusts were proposed to jump was illuminated using four LED illuminators (RODER SRL, Oglianico, TO, Italy) that emit 420 lm each at k = 628 nm. Red light corresponds to the maximum absorption frequency of the camera, and it does not impair the visual apparatus of the locusts since these insects are blind to light of such wavelength (Briscoe and Chittka, 2001). Selected videos were edited with NAC HSSC Link software (NAC Image Technology) to extract the takeoff phase part from the whole video, and the Computer Vision Toolbox of MATLAB 2017b (The MathWorks, Inc., Novi, MI, USA) was then used to track object movement.

Both Bold and “*” is to emphasize that value is smaller than 0.05, and the effect is significant for tested parameter.

Successful jumps were picked out from all recorded jumps, and the centroids of locusts were selected and tracked using the Computer Vision Toolbox. By means of the centroid trajectory (in pixels over time) and the corresponding scale information provided by the filter paper, the displacement was calculated in millimeters. Polynomial regression methods were then used to analyze trajectories and instantaneous velocities, then the following variables were calculated: (1) takeoff time, i.e., the time between the moment just before jumping and the end of takeoff phase when hind legs leave the ground; (2) takeoff angle, which is equal to the inclination angle that fits, under first order approximation, the centroid trajectory during takeoff phase; (3) takeoff velocity, which is the instantaneous velocity when loosing contact with ground; (4) average acceleration during takeoff phase, which is the ratio between the takeoff velocity and the takeoff time.

**E** and ***v***are material properties; in particular **E**, the Young Modulus, is the extensional rigidity. Starting from these properties, it is possible to derive some quantities that characterize the particular objects in the experiments, depending on the geometries (mainly on thickness). These are *K*_*ext*_, the extensional stiffness per unit length (in *x* or *y* direction) and *K*_*bend*_, the flexural stiffness per unit length (in *x* or *y* direction).

For aluminum plate:

(1) $$\begin{array}{l} \left\{ \begin{array}{c} {K_{ext}}_{al}=\frac{E_{al}h_{al}}{1-\nu_{al}^{2}}=\frac{7.2\cdot 10^{4}\frac{N}{mm^{2}}\cdot 3mm}{1-0.334^{2}}\approx 2.43\cdot 10^{5}\frac{N}{mm}\\ {K_{bend}}_{al}=\frac{E_{al}h_{al}^{3}}{12\left(1-\nu_{al}^{2}\right)}=\frac{7.2\cdot 10^{4}\frac{N}{mm^{2}}\cdot {\left(3mm\right)}^{3}}{12\left(1-0.334^{2}\right)}\approx 1.82\cdot 10^{5}Nmm \end{array}\right. \end{array}$$

For one-layer silicone membrane:

(2) $$\begin{array}{l} \left\{ \begin{array}{c} {K_{ext}}_{one-lay-sil}=\frac{E_{sil}h_{one-lay-sil}}{1-\nu_{sil}^{2}}\approx 1.33\frac{N}{mm}\\ {K_{bend}}_{one-lay-sil}=\frac{E_{sil}h_{one-lay-sil}^{3}}{12\left(1-\nu_{sil}^{2}\right)}\approx 4.44\cdot 10^{-3}Nmm\; \end{array}\right. \end{array}$$

For three-layer silicone membrane:

(3) $$\begin{array}{l} \left\{ \begin{array}{c} {K_{ext}}_{three-lay-sil}=\frac{E_{sil}h_{three-lay-sil}}{1-\nu_{sil}^{2}}\approx 4\frac{N}{mm}\\ {K_{bend}}_{three-lay-sil}=\frac{E_{sil}h_{three-lay-sil}^{3}}{12\left(1-\nu_{sil}^{2}\right)}\approx 1.20\cdot 10^{-1}Nmm \end{array}\right. \end{array}$$

As evident, in case of aluminum plate the extensional and flexural stiffnesses are comparable, while in case of silicone substrates the flexural stiffnesses values are orders of magnitude lower than the extensional stiffnesses. As a consequence, in the mathematical model proposed Appendix, flexural stiffness will be neglected, and thus omitted, in the part regarding membrane modeling.

### Statistical Analysis

The influence of substrate's compliance was separately analyzed for each of the previously described parameters, i.e., takeoff time, takeoff angle, takeoff velocity and average acceleration. A generalized linear model *y* = β*X*+ε was adopted, where *y* is the vector of the observations with normal distribution (takeoff time and takeoff angle), β is the incidence matrix linking the observations to fixed effects, *X* is the vector of fixed effects (i.e., substrates' compliance) and ε is the vector of the random residual effects. All data have been analyzed by using R software v3.6.1.

## Results

In total 226 locusts' jumps were taken into account, including 65 jump from rigid substrate (29 males and 36 females), 90 jump from compliant substrate (64 males and 36 females), and 71 jump from most compliant substrate (35 males and 36 females). They were analyzed with the abovementioned methods. The results are illustrated within the following subsections.

### The Effect of Substrates on Locusts' Jumping Performance

Jumping performances of locusts are strongly connected with body weight and, as concerns locusts tested in the present study, females (Mean = 2.11, Std error = 0.02) are significant heavier than males (Mean = 1.47, Std error = 0.02, *F*_1, 225_ = 706.37, *P* < 0.001). The effect of gender on dependent variables was tested using one-way repeated ANOVAs. There are some significant differences between males and females if dependent variables like takeoff time (*F*_1, 225_ = 25.50, *P* < 0.001) and average acceleration (*F*_1, 225_ = 7.47, *P* < 0.05) are considered, while no significant differences were encountered for the takeoff angle (*F*_1, 225_ = 0.0046, *P* = 0.95) and takeoff velocity (*F*_1, 225_ = 2.01, *P* = 0.16). The ANCOVAs revealed significant interaction effects between animal body weight and substrate compliance for two variables, the takeoff velocity and the average acceleration (Table 2). The takeoff velocity of jumps on most compliant substrate (Mean = 1.37, std = 0.084) are significantly smaller than compliant (Mean = 2.07, std = 0.081) and rigid (Mean = 1.87, std = 0.089, *F*_2, 225_**=**18.17, *P* < 0.001) substrate. The average acceleration of jumps on compliant (Mean = 59.14, std = 2.45) substrate significantly higher than that of rigid substrates (Mean = 48.7, std = 2.68), and the acceleration of jumps on rigid substrate is significantly higher than that of most complaint substrate (Mean = 35.8, std = 2.54, *F*_2, 225_**=**29.96, *P* < 0.001). Based on this, the effect of compliance on jumping performances of female and male locusts are evaluated individually, using ANCOVA and considering body weight as a covariant.

**Table 2:** *F*-value and associated significance levels for one-way repeated-measurement ANCOVA for jump variables across three treatments: rigid, compliant and most compliant substrates, with body weight as a covariate, Different letters above each column indicate significant differences (*P* < 0.05).

**Variables**	**Compliance level**		**Mass**		**Compliance level** **×** **Mass**	
	**F_2**, **225_**	**P**	**F_2**, **225_**	**P**	**F_2**, **225_**	**P**
Takeoff time	13.69	<0.001*	15.86	<0.001*	0.73	0.48
Takeoff angle	22.39	<0.001*	6.07	0.01*	0.12	0.89
Takeoff velocity	18.17	<0.001*	0.33	0.57	4.11	0.02*
Acceleration	29.96	<0.001*	4.86	0.03*	5.72	0.003*

**Figure 2:**
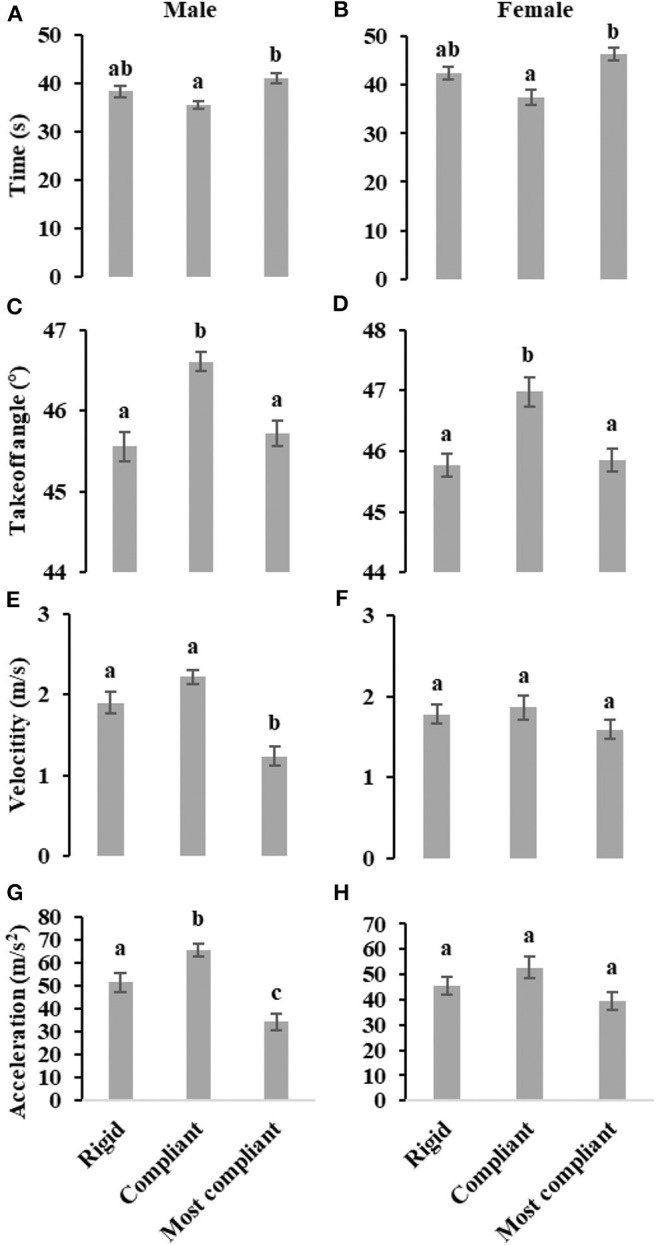
Effect of three levels of substrates compliance on takeoff phase time **(A,B)**, takeoff angle **(C,D)**, takeoff velocity **(E,F)** and average acceleration during takeoff phase **(G,H)** of male and female locusts, individually evaluated using ANCOVA while considering body weight as a covariate. Different letters above each column indicate significant differences (*P* < 0.05). Whiskers represent standard errors.

As illustrated in Figures 2A,B, the takeoff time on most compliant substrate is significantly longer than takeoff time on compliant substrate, both for male (*F*_2, 127_ = 9.27, *P* < 0.001) and female (*F*_2, 97_ = 7.98, *P* < 0.001) locusts. For both males (*F*_2, 127_ = 16.67, *P* < 0.001) and females (*F*_2, 97_ = 8.06, *P* < 0.001), the takeoff angle on compliant substrate is significant greater than takeoff angles on rigid and most compliant substrate, as shown in Figures 2C,D. The takeoff velocity of male locusts on most compliant substrate is significantly lower than on rigid and compliant substrates (*F*_2, 127_ = 21.65, *P* < 0.001), while for female locusts, there is no significant difference (*F*_2, 97_ = 1.20, *P* = 0.30), as illustrated in Figures 2E,F. In addition, male locusts display a relative lower takeoff velocity on compliant substrate (*F*_1, 90_ = 5.24, *P* < 0.05). Considering the average acceleration during takeoff phase for male locusts (*F*_2, 127_ = 23.17, *P* < 0.001), the mean acceleration on compliant substrate is significantly greater than that of most compliant substrate, and the mean acceleration on rigid substrate is significantly higher than that on most compliant substrate. In contrast, there is no significant difference in the average acceleration for female locusts (*F*_2, 97_ = 2.74, *P*=0.07), as shown in Figures 2G,H.

### The Recoil of the Compliant Substrates

**Figure 3:**
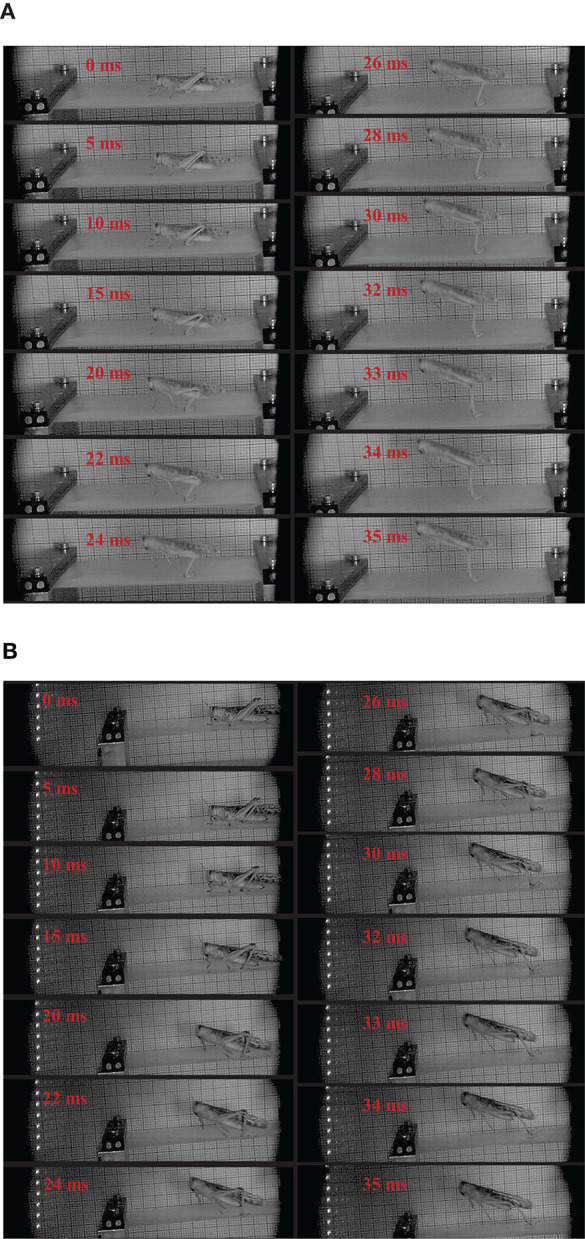
The sequential pictures during takeoff from compliant substrates for two different locusts. The moment with first leg movement was noticed at 0 ms. The number on each image indicates the time after 0 ms. In the jumping sequence reported in the figure, the takeoff phase last 35 ms, the moment a locust leaves the substrates is defined as 35 ms. **(A)** The sequential pictures during takeoff from compliant substrates. **(B)** The sequential pictures during takeoff from most compliant substrates.

**Figure 4:**
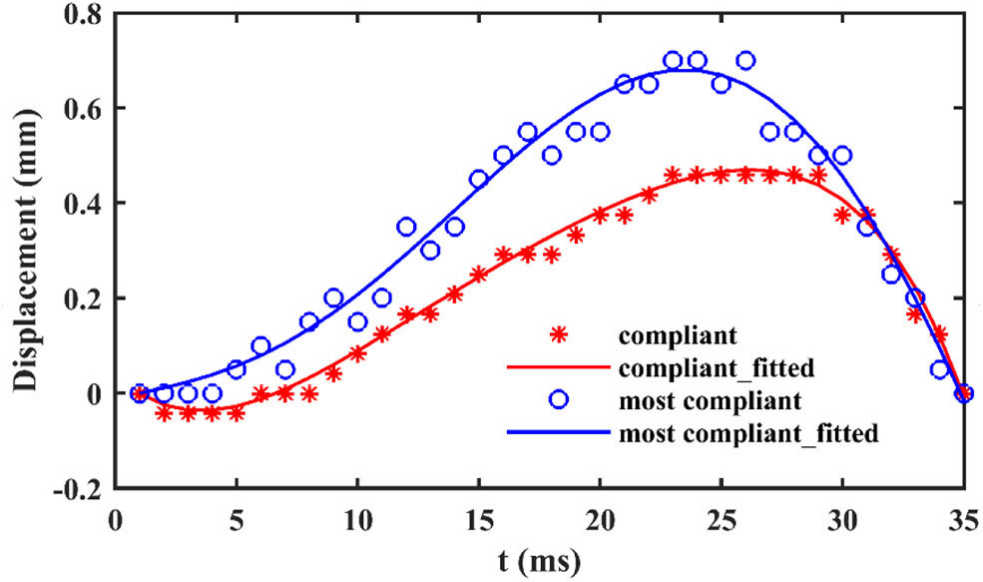
The vertical displacement over time of compliant and most compliant substrates in contact point.

When locusts jump from rigid substrate, like the aluminum test bench in our test, the deformation of substrate can be ignored. In contrast, there are notable deformations during the takeoff phase, when locusts jump from the other two more compliant substrates, as shown in Figure 3. During the takeoff phase, the deformation of the compliant substrate increases and then descends after reaching a maximum (It is clearer in the attached videos, downloading links are listed in Supplementary Materials), as illustrated in Figure 4. Before locusts leave the substrates, there is an obvious recoil phase for the compliant substrates. The maximum deformation of compliant substrate during takeoff is around 0.47 mm, and this value for most compliant substrate during takeoff is around 0.69 mm.

## Discussion

In terms of relative performances, histograms about time, velocity, acceleration and takeoff angle in Figure 2 show that jumping performances vary less in case of female locusts, meaning that females own better adaptability to different levels of substrate's compliance, while, in terms of absolute performances, it is evident that males can execute more powerful jumps (greater absolute values of velocity, takeoff angle, acceleration and smaller time) with respect to females if rigid and compliant substrates are considered. This aspect is likely due to males' smaller weight. If most compliant substrate is considered, instead, females highlight greater absolute performances, confirming once more their major adaptation capability to substrates of different compliance. In other words, although males benefit from less weight, they have so much less adaptability with respect to females to be precluded from reaching equal absolute performances when a too compliant substrate is selected.

So far, relative and absolute performances have been compared between male and female locusts. Shifting now to global jumping performances of locusts in general (without gender differentiation), males and females share the same performance trend throughout the three different kind of substrates: best performances on compliant substrate, intermediate performances on rigid substrate and worst performances on most compliant substrate. This means that locusts are in general able to keep advantage from the elastic action (spring effect) of a moderately compliant substrate by increasing their jumping performances, while they are subjected to excessive energy losses in substrate's deformation when compliance reaches higher values.

**Figure 5:**
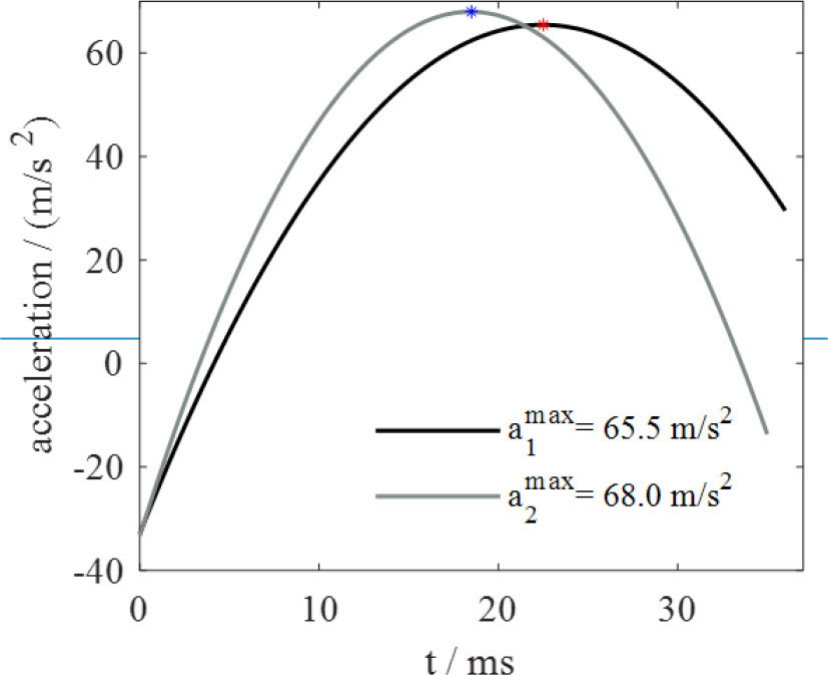
Acceleration trend during takeoff phase: *a*_1_ on a rough foam surface, *a*_2_ on a smooth acrylic surface.

As regards jumps from the compliant and most compliant substrates, the deformation follows an ascending and then descending trend during the takeoff phase. By observing the acceleration trend in Figure 5, taken from a previous work (Mo et al., 2019) of the authors of this text, similarities with the substrate deformation trend are evident. Since contact force is proportional to acceleration, it is possible to conclude that the deformation of silicone substrates and the deformation of contact force follow similar trends. These observations suggest that the capability to adapt to substrates of different compliances is correlated to that force trend. It seems that locust, thanks to their long legs that allows them to longer keep in touch with ground, are able to follows the natural trend of the substrates recoil, rather than hamper it, and this permits to these insects to exploits the so called spring effect in order to waste a minimum amount of energy. In fact, it's not a coincidence that the peak of the acceleration, and so of the force, in Figure 5 and the maximum vertical displacement of the substrate (corresponding to the maximum of the recoil phase) in Figure 4 occur at very close instants of time. The substrates tested in Mo et al. (2019) are rough foam board (recalling the compliant substrate) and smooth acrylic substrate (recalling rigid substrate).

**Figure 6:**
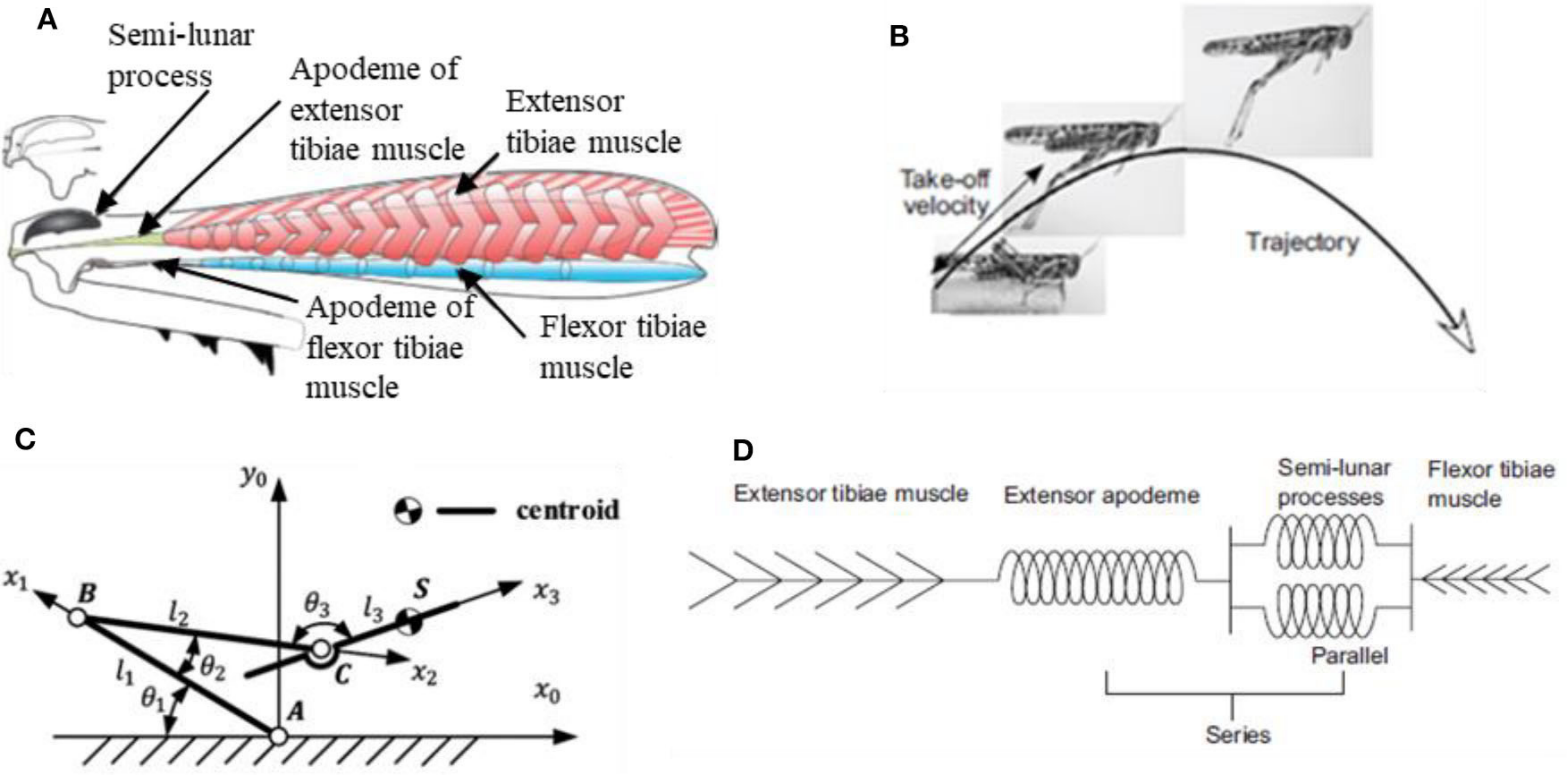
**(A)** Mechanical diagram of the interactions between the extensor tibiae muscle, its apodeme and the semilunar processes: during the contraction just before jumping, the apodeme pulls on and distorts the front part of the femoro-tibial joint, bending the semi-lunar processes (Rogers et al., 2016); **(B)** configuration's variation from the steady stare to the jumping state (Rogers et al., 2016); **(C)** theoretical model of locust jumping cinematics (Mo et al., 2019); **(D)** lumped parameter model of muscles' arrangement in locust jump (Rogers et al., 2016).

The strategic role played by the specific jumping technique is also confirmed by what observed in tree frogs (Knight, 2015). Locusts and tree frogs, in fact, share the same trend in contact force during takeoff and the same particular legs cinematics in jumping, that is referred to as “catapult mechanism.” This latter deserves a closer look since it's likely the real key to the success of these animals in adapting to different substrates. The pattern for jumping in animals that exploit catapult mechanism always present two fundamental phases: in the first phase elastic energy is accumulated in the muscle fibers; in the second phase the elastic energy is explosively released to power the jump. In the specific case of locusts (Figure 6), the jumping motor pattern consists of three phases (Rogers et al., 2016): the cocking phase, the co-contraction phase and the jump phase (or triggering phase). During the cocking phase, the hind tibiae are totally flexed and blocked into position against the hind femora. Then, during the co-contraction phase, the extensor tibiae muscle and its antagonist flexor tibiae muscle of each leg contract together, but no movement occurs; instead, the semi-lunar processes and the extensor apodeme are both steadily deformed to store the energy produced by the prolonged contraction of muscles (they act as torsional springs). Finally, during the jump phase, the flexor-tibiae motor neurons are inhibited, allowing the tibiae to move; the energy that was stored in the semi-lunar processes and extensor apodemes is suddenly released providing power for the takeoff.

To better understand, Figure 6 provides some schematic representations of locusts' anatomy and cinematic. In particular, Figure 6A (Rogers et al., 2016) shows a representation of the internal anatomy of a hind femur, characterized by a massive, pennate extensor tibiae muscle and a very small flexor tibiae muscle, while Figure 6B (Rogers et al., 2016) shows how the leg configuration varies from the steady phase to the jumping phase. Figure 6C (Mo et al., 2019) represents a theoretical model of locust jumping cinematic: the body is simplified as rigid; the centroid is located in point S; femur is connected with the body by means of joint C. Femur and tibiae were simplified as rigid bars and the knee joint was simplified as hinge B. Tarsus and ground are simplified as one part and the joint between tarsus and tibiae is simplified as hinge A. θ_1_, θ_2_, θ_3_ represent the angles between the links separately, *l*_1_, *l*_2_, *l*_3_ represent the length of femur bar AB, tibiae bar BC and the length between point C and centroid S, respectively. For more details on how reference systems are chosen and for an accurate mathematical formulation, the interested reader is invited to directly consult the reference (Mo et al., 2019). Finally, Figure 6D (Rogers et al., 2016) shows a lumped parameter modelling of the locust's leg cinematic, highlighting how the extensor tibiae muscles operate in parallel with each other but in series with the extensor apodeme.

Another analogy in animal world is found with gibbons about the takeoff time trend. Gibbons, in fact, tend to move much slower when jumping from highly compliant poles, in the attempt to minimize the pole's deflection and, thus the potential energy losses (Channon et al., 2011).

In general, jumps from compliant substrates present several challenges, and could have detrimental impact on jumping performances, mainly the takeoff velocity and the jumping stability. During locusts' jumps, the substrate deforms under the application of contact force, making balance maintenance quite complex for locusts. The body angle relative to horizontal direction keep consistent during the takeoff phase and the following in-air phase.

In conclusion, locusts own an excellent capability to adapt to different substrate compliance levels, exploiting the recoil phase of the substrate to regain part of the energy lost in the deformation of the substrate itself. If the compliance is moderate, it can even constitute an advantage for locusts' jumping activity, while for high levels of compliance performances decrease. Anyway, even on most compliant substrate locusts showed the ability to limit energy wasting, and so the drop in performances. No instability phenomena were detected.

Most existing jumping robots ignores the compliance of the substrates; they are supposed to jump from a rigid substrate, while the compliance of the substrate cannot be simplified as rigid directly. There is no related research on robots' jumping performance on compliant substrates, nor the strategies that robots use to adapt their jumping performance to compliant substrates. This research can give some insights for similar research on jumping robots. The strategy used by locusts to adapt to substrates' compliance can be mimicked by bio-inspired jumping robots. In particular, in order to endow their products with the capability to adapt to substrate of different compliances, roboticists may implement control algorithms that reproduce the particular force trend highlighted above. Of course, also robots' legs conformation should be similar to that of locust, in order to facilitate the task.

To the best of authors' knowledge, this is the first report on the effect of substrate's compliance on locusts jumping performances. These findings improve the understanding of the jumping mechanism in locusts, as well as can be used to develop artifact outperforming current jumping robots in unstructured scenarios.

## Data Availability Statement

The datasets generated for this study are available on request to the corresponding author.

## Ethics Statement

This research adheres to the guidelines for the treatment of animals in behavioral research and teaching (ASAB/ABS, 2014), the country (Italy) where the experiments were performed (D.M. 116192) (Listed, 2008), and the European Union regulations (European Commission, 2007). All experiments are behavioral observations, and no specific permits are required in the country where the experiments were conducted.

## Author Contributions

XM and DR conceived and designed the study. XM and MM performed the experiment. XM and WG analyzed the data. XM, DR, WG, and CS interpreted results. All authors wrote and contributed to the final version of the manuscript and approved the submission.

## Conflict of Interest

The authors declare that the research was conducted in the absence of any commercial or financial relationships that could be construed as a potential conflict of interest.

## Supplementary Material

Supplementary Video 1 Takeoff from compliant substrate.

Supplementary Video 2 Takeoff from most compliant substrate.
