# Supplementary material for: Effect of Substrates' Compliance on the Jumping Mechanism of Locusta migratoria
Source: Front Bioeng Biotechnol. 2020 Jul 6;8:661. doi: 10.3389/fbioe.2020.00661 (PMC7381386; doi:10.3389/fbioe.2020.00661)
Supplement: Supplementary file 1 [file Presentation_1.pdf]

Supplementary Materials

To prepare the ground for future studies in which the experimental results might be compared with theoretical ones, a mathematical model is briefly illustrated. Of course, the model can be further developed and, for instance, employed to estimate the amount of energy recovered by locusts in the recoil phase of the substrate and the amount that is lost, thus obtaining an estimate of the efficiency of the jumping technique.

Mathematical modeling of the test bench

As concerns tests on the aluminum substrate, the jumping test bench can be modeled as a plate subjected to a short pulse load while, for the tests on the silicone substrates, a non-linear model for membranes subjected to an impulsive load is more appropriate. Figure 4 will be taken as reference.

Figure A1: Thin plate with reference system and constraints

Case I: plate with short pulse load

According to this work's authors, a valid reference is [1], in which the dynamic response of orthotropic plates is analyzed through the modal superimposition approach. Firstly, the effect of damping will not be considered; it will be commented at a later time. Below each equation for orthotropic material taken from [1], the authors of this work will add, when possible, the corresponding equation for an isotropic material, that is approximatively the case of aluminum

Following the Classical Plate Theory, the bending dynamics of plates is given by:

$$\begin{cases} D_{11}\frac{\partial^4 w}{\partial x^4} + 2(D_{12} + 2D_{66})\frac{\partial^4 w}{\partial x^2 \partial y^2} + D_{22}\frac{\partial^4 w}{\partial y^4} + m\frac{\partial^2 w}{\partial t^2} = q(x,y,t)\ orthotropic \\ \frac{1}{E}\left(\frac{\partial^4 w}{\partial x^4} + \frac{\partial^4 w}{\partial y^4}\right) + \left(\frac{4}{E} - \frac{2v}{E}\right)\frac{\partial^4 w}{\partial x^2 \partial y^2} + m\frac{\partial^2 w}{\partial t^2} = q(x,y,t)\ isotropic \end{cases} \quad \text{(A1)}$$

Where w is the transverse displacement, q is the applied pressure loading, m is the mass per unit area, and $D_{ij}s$ are the bending rigidities of the orthotropic plate. According to the superimposition approach, the transverse displacement can be expanded in the double series

$$w(x,y) = \sum_{i=1}^{m}\sum_{j=1}^{n}\alpha_{ij}(t)\varphi_{ij}(x,y) \quad \text{(A2)}$$

in which the various $\alpha_{ij}(t)$ are obtained by solving a system of $m \times n$ uncoupled equations of the form:

$$m_{ij}\ddot{\alpha}_{ij} + k_{ij}\alpha_{ij} = f_{ij}(t) \quad \text{(A3)}$$

For the particular case of this work, the rectangular plate, the stiffness coefficients are expressed by

$$k_{ij} = \int_0^a \int_0^b L_{ij} dxdy \tag{A4}$$

With

$$\begin{cases} L_{ij} = D_{11}\left(\frac{\partial^2 \varphi_{ij}}{\partial x^2}\right)^2 + D_{22}\left(\frac{\partial^2 \varphi_{ij}}{\partial y^2}\right)^2 + 2D_{12}\frac{\partial^2 \varphi_{ij}}{\partial x^2}\frac{\partial^2 \varphi_{ij}}{\partial y^2} + 4D_{66}\left(\frac{\partial^2 \varphi_{ij}}{\partial x \partial y}\right)^2 \; orthotropic \; [55] \\ L_{ij} = \frac{1}{E}\left[\left(\frac{\partial^2 \varphi_{ij}}{\partial x^2}\right)^2 + \left(\frac{\partial^2 \varphi_{ij}}{\partial y^2}\right)^2\right] - \frac{2\nu}{E}\frac{\partial^2 \varphi_{ij}}{\partial x^2}\frac{\partial^2 \varphi_{ij}}{\partial y^2} + \frac{4}{G}\left(\frac{\partial^2 \varphi_{ij}}{\partial x \partial y}\right)^2 \; isotropic \end{cases} \tag{A5}$$

The modal masses and forces, instead, are defined by

$$m_{ij} = m \int_0^a \int_0^b \varphi_{ij}^2 dxdy \tag{A6}$$

and by

$$f_{ij}(t) = \int_0^a \int_0^b q(x,y,t)\varphi_{ij}(x,y) dxdy \tag{A7}$$

In the general case the plate is loaded by distributed pressure $p = \bar{p}(x,y)h(t)$ where $h(t)$ is the function describing the shape of the pulse in time. So, according to equation, $f_{ij}(t) = F_{ij}h(t)$ with

$$F_{ij} = \int_0^a \int_0^b \bar{p}(x,y)\varphi_{ij}(x,y) dxdy \tag{A8}$$

Since locusts exert force on the ground in a very short time, it is possible to use the impulsive approximation:

$$\alpha_{ij_{pulse}}(t) \approx \frac{1}{m_{ij}\omega_{ij}}\left\{I \sin(\omega_{ij}t) - M\omega_{ij}\cos(\omega_{ij}t)\right\} \tag{A9}$$

where $I = \int_0^t f(\varepsilon)d\varepsilon$ is the applied impulse, representing the area under the force versus time curve and $M = \int_0^t \varepsilon f(\varepsilon)d\varepsilon$ is the first moment of that area.

Effect of damping

In [1] also the effect of damping is accounted for using modal approach. In that way, the modal equations are rewritten as

$$\ddot{\alpha} + 2\eta\omega\dot{\alpha} + \omega^2\alpha = f(t) \tag{A10}$$

where η is the modal damping ratio. As it increases, oscillations are damped out and the response tends to a quasi-static one, characterized by $\alpha = f(t)/\omega^2$.

Case II: non-linear damped model for membranes

In this case a valid reference is [2], in which a solution for the non-linear damped forced vibration of a prestressed orthotropic rectangular membrane is provided by using the Krylov-Bogolubov-Mitropolsky (KBM) perturbation method. Some little differences exist between the particular case studied in and the case object of this work. In fact, the membrane in [2] has all the four edges fixed and prestressed while the silicone membrane in locust jumping experiments has two opposite sides fixed and prestressed while the other two are totally free. Moreover, in the impulsive load to the membrane is given by a pellet that impacts the mundane with v_0 velocity, while on the silicone the impulsive load consists of the muscular force of locusts, suddenly exerted in a very short time. Despite these little differences, [2] is taken as reference anyway, mainly for two reasons: 1) in the experiments locust are placed in the center of the silicone membrane, far away from the free edges, that can thus be considered with negligible vertical displacements and rotations with respect of the central portion of the membrane; 2) As the authors of [2] state themselves, their study constitutes a valid basis for the dynamic study of membrane structures in general.

According to von Kármán large deflection theory and D'Alembert's principle [3], the dynamic governing equations are:

$$\begin{cases} \rho\frac{\partial^2 w}{\partial t^2} + c\frac{\partial w}{\partial t} - (N_x + N_{0x})\frac{\partial^2 w}{\partial x^2} - (N_y + N_{0y})\frac{\partial^2 w}{\partial x^2} = p(x,y,t) \\ \frac{1}{E_1 h}\frac{\partial^2 N_x}{\partial y^2} - \frac{v_2}{E_2 h}\frac{\partial^2 N_y}{\partial y^2} - \frac{v_1}{E_1 h}\frac{\partial^2 N_x}{\partial x^2} + \frac{1}{E_2 h}\frac{\partial^2 N_y}{\partial x^2} - \frac{1}{Gh}\frac{\partial^2 N_{xy}}{\partial x \partial y} = \left(\frac{\partial^2 w}{\partial x \partial y}\right)^2 - \frac{\partial^2 w}{\partial x^2}\frac{\partial^2 w}{\partial y^2} \end{cases} \tag{A11}$$

Where ρ is the membrane areal density, c is the viscous damping, N_x and N_y are the additional tensions in x and y directions, N_{0x} and N_{0y} the initial tensions in x and y directions, N_{xy} is the in-plane shear force, $w(x,y,t)$ is the vertical displacement, h is the membrane's thickness, E_1, E_2 and v_1, v_2 are respectively the Young's moduli and the Poisson's rations x and y directions and G is the shear modulus. Since the silicone membrane was manufactured by uniformly spreading a thin layer on a stainless-steel sheet, it is possible to assume that the mechanical properties in the x and y directions are the same and that they differ in the direction of the thickness. So, by posing $E_1 = E_2 = E$ and $v_1 = v_2 = v$, for the silicone membranes the following holds:

$$\begin{cases} \rho\frac{\partial^2 w}{\partial t^2} + c\frac{\partial w}{\partial t} - (N_x + N_{0x})\frac{\partial^2 w}{\partial x^2} - N_y\frac{\partial^2 w}{\partial x^2} = p(x,y,t) \\ \frac{1}{Eh}\left(\frac{\partial^2 N_x}{\partial y^2} + \frac{\partial^2 N_y}{\partial x^2}\right) - \frac{v}{Eh}\left(\frac{\partial^2 N_y}{\partial y^2} + \frac{\partial^2 N_x}{\partial x^2}\right) - \frac{1}{Gh}\frac{\partial^2 N_{xy}}{\partial x \partial y} = \left(\frac{\partial^2 w}{\partial x \partial y}\right)^2 - \frac{\partial^2 w}{\partial x^2}\frac{\partial^2 w}{\partial y^2} \end{cases} \tag{A12}$$

In the end, the solution found in [2] is:

$$w(x,y,t) = \sum_{m=1}^{\infty}\sum_{n=1}^{\infty} \sin\frac{m\pi x}{a} \sin\frac{n\pi y}{b} * \left(De^{0.5\alpha_2 \varepsilon t} \cos\left(\left(\omega_0 - \frac{3\alpha_1 \varepsilon D^2 e^{\alpha_2 \varepsilon t}}{8\omega_0} \right) t + \frac{\pi}{2} \right) \right) \quad \text{(A13)}$$

In order not to excessively weight down the text, the complete definition of all parameters in equation will be omitted. The interested reader can find it in [2] with great attention to details.

Attached videos:

Attached are two takeoff phase videos

Video 1: Takeoff from compliant substrate

Video 2: Takeoff from most compliant substrate

References

[1] Abrate, S. (2007, January). Transient response of beams, plates, and shells to impulsive loads. In ASME 2007 International Mechanical Engineering Congress and Exposition (pp. 107-116). American Society of Mechanical Engineers Digital Collection.

[2] Liu, C. J., Zheng, Z. L., Yang, X. Y., & Zhao, H. (2014). Nonlinear damped vibration of pre-stressed orthotropic membrane structure under impact loading. International Journal of Structural Stability and Dynamics, 14(01), 1350055.

[3] Lewicka, Marta, L. Mahadevan, and Reza Pakzad (2010). The Von Kármán equations for plates with residual strain. arXiv preprint arXiv:1002.2252.
